# Assessment for bone health in patients with differentiated thyroid carcinoma after postoperative thyroid-stimulating hormone suppression therapy: a new fracture risk assessment algorithm

**DOI:** 10.3389/fendo.2023.1286947

**Published:** 2023-11-21

**Authors:** Huiran Jia, Wei Qu, Xiaoting Cai, Meiye Li, Ying Qian, Zhaoshun Jiang, Zongjing Zhang

**Affiliations:** ^1^ Endocrinology Department, Postgraduate Training Base of Jinzhou Medical University, Jinzhou, Liaoning, China; ^2^ Endocrinology Department, The 960th Hospital of the People’s Liberation Army Joint Logistics Support Force, Jinan, China

**Keywords:** differentiated thyroid cancer, fracture risk assessment tool (FRAX), osteoporosis, TSH suppression therapy, bone mineral density (BMD)

## Abstract

**Purpose:**

The fracture risk assessment tool (FRAX) is used to assess the 10-year risk of major site and hip fractures; however, whether this tool can be applied to patients receiving levothyroxine-based thyroid-stimulating hormone (TSH) suppressive therapy for postoperative differentiated thyroid cancer (DTC) patients is yet to be clarified.

**Methods and design:**

A total of 64 patients with DTC following thyroidectomy and oral levothyroxine for TSH suppression therapy and 30 gender- and age-matched controls were collected. The fracture risk was compared between the affected groups with different TSH levels. FRAX was used to calculate the fracture risk with and without bone mineral density (BMD). The TSH level was converted to an age-weighted score to estimate the fracture risk of postoperatively differentiated thyroid cancer patients. The sensitivity, specificity, and area under the AUC curve of the traditional FRAX and the new algorithm for osteoporosis diagnosis were compared. The dual-energy X-ray bone mineral density measurement T score was used as the gold standard to diagnose osteoporosis.

**Results:**

There were 24 patients in the T ≥ −1–2.5 group, 23 in the −2.5 < T < −1 group, and 17 in the T ≤ −2.5 group. The T score of BMD in the disease group was significantly lower than that in the control group (*p* < 0.05). The risk of MOF and hip fracture without a T score were significantly different under various TSH levels (*p* < 0.05). The area under the curve (AUC) of FRAX without BMD for predicting major osteoporotic fractures (PMOF) and major hip fractures (PHF) was 0.694 and 0.683, respectively. The cutoff values were 2.15% and 0.25%, respectively. The AUC of FRAX with BMD for PMOF and PHF was 0.976 and 0.989, respectively, and the cutoff values were 4.15% and 1.1%, respectively. The AUC of FRAX without BMD for PMOF and PHF was 0.708 and 0.72, respectively, and the cutoff values were 5.5% and 1.55%, respectively.

**Conclusions:**

FRAX is suitable for postoperative DTC patients after TSH suppressive therapy. In the absence of BMD, TSH weighted by age can improve the specificity of FRAX in the diagnosis of osteoporosis in this population.

## Introduction

Osteoporosis is a metabolic bone disease characterized by loss of bone mass and microstructure destruction of bone tissue. It has a negative impact on the lives of patients, causing disability, chronic pain, and increased mortality (1, 2), and a specific impact on the quality of life and the mental health of the elderly (3–5). The cost of osteoporosis imposes a significant economic burden worldwide (6). A 2016 study revealed that the total cost of osteoporosis in Canada exceeded $4.6 billion, an 83% increase from the 2008 estimate (7). Secondary osteoporosis is diagnosed when bone fragility is caused by a disease, drug, or nutritional deficiencies. The several causes of secondary osteoporosis include diabetes, thyroid and parathyroid disease, malabsorption, inflammatory bowel disease (IBD), irritable bowel syndrome(IBS), nutritional causes, drugs, infection, anemia, malignancy, inflammatory arthritis, systemic lupus erythematosus (SLE), smoking, and genetic factors (8). The present study focused on osteoporosis caused by TSH suppression therapy postoperatively. DTC is one of the most common endocrine tumors (9). Patients with DTC who underwent thyroidectomy or iodine ablation need TSH suppression therapy (serum TSH level < 0.5 mIU/L) with levothyroxine to minimize TSH-mediated tumor growth and recurrence (10). TSH suppression significantly increased the risk of postoperative osteoporosis in patients with low- to moderate-risk DTC but did not change tumor recurrence (11). Lin et al. showed a high risk of osteoporosis in differentiated thyroid cancer patients treated with levothyroxine. The cumulative duration of levothyroxine use for > 2,751 days (equivalent to 7.53 years) was associated with a 3.34-fold high risk of osteoporosis (12).

FRAX is a fracture risk assessment tool launched by the World Health Organization (WHO) in 2008. Fracture risk factors, such as gender, age, and height, are included in calculating the 10-year risk of hip and major fractures (13). FRAX has been widely used and included in the osteoporosis assessment guidelines in many countries, and FRAX with BMD has been proven to be more effective than BMD alone in identifying high-risk individuals (14, 15).

However, the risk factors for TSH suppression following thyroidectomy were not included in the FRAX system. The present study aimed to evaluate the changes in BMD in patients with differentiated thyroid cancer after TSH suppression therapy, the applicability of FRAX in this population, and develop a new, more convenient, and faster FRAX algorithm for this population instead of BMD measurement.

## Materials and methods

### Study design and population

A total of 64 patients with DTC following thyroidectomy and oral levothyroxine for TSH suppression therapy in the 960th Hospital of the People’s Liberation Army from October 2021 to May 2022 were collected retrospectively. In addition, 30 cases comprising the control group were matched by age, sex, and major concomitant disease (for example, diabetes) by approximately 2:1. All patients were > 20 years old, and their BMD of the hip and lumbar spine was measured. Inclusion criteria were as follows (1): patients with postoperative TSH suppression therapy for at least 6 months (2); unable to carry out normal activities and adhere to follow-up. Patients with diseases affecting bone metabolisms, such as thyroid and parathyroid dysfunction and other neoplastic diseases, using drugs affecting bone metabolism, such as calcitonin and bisphosphonates, and being unwilling to cooperate, were excluded from the study. Those who were currently pregnant or planning to become pregnant were also excluded. Patients’ general information, including gender, age, height, weight, operation time, history of brittle fracture, rheumatoid arthritis, medical history, parent history of hip fractures, smoking, drinking, hormone use, secondary osteoporosis, and history of thyroid and parathyroid dysfunction, was collected using a questionnaire survey. The fracture risk was compared between the affected group and the control group and between the affected group with different TSH levels. The sensitivity, specificity, and area under the AUC curve of the traditional FRAX and the new algorithm for osteoporosis diagnosis were compared. This study was approved with informed consent by the Ethics Committee of the Hospital.

### BMD and FRAX

BMD was measured at the hip using dual-energy X-ray absorptiometry (Lunar-Prodigy, GE, USA). Herein, we used three methods to calculate the risk of fracture in the postoperative population for DTC, yielding three datasets. First, the Chinese mainland model was selected from https://www.sheffield.ac.uk/FRAX/tool, and the relevant information (including age, gender, low BMD, low body mass index (BMI) ≤ 19 kg/m^2^, previous fragility fracture history, parental hip fracture history, glucocorticoid treatment history, smoking, excessive alcohol consumption, and rheumatoid arthritis) was entered as demanded. The predictive value of 10-year major osteoporotic fracture (PMOF without BMD) and hip fracture (PHF without BMD) was calculated. Second, in addition to the above information, we entered the T score to recalculate the fracture risk of this population (PMOF with BMD and PHF with BMD). For the third group of data, the age information was added according to the TSH-weighted scoring method while entering the basic information, and the BMD was not entered, while the other information was the same as before. According to the different levels of TSH, the age information was added extra based on the age of the patient. The age of TSH of < 0.008 µIU/mL (less than the detectable value) was increased by 20 years, the age of TSH of < 0.1 µIU/mL was increased by 15 years, 0.1 µIU/mL < TSH ≤ 0.2 µIU/mL was increased by 10 years, and 0.2 µIU/mL < TSH ≤ 0.3 µIU/mL was increased by 8 years. 0.3 µIU/mL < TSH ≤ 0.4 µIU/mL plus 6 years, 0.4 µIU/mL < TSH ≤ 0.5 µIU/mL plus 4 years, 0.5 µIU/mL < TSH ≤ 2 µIU/mL plus 2 years, and TSH > 2 µIU/mL years were included in the patients’ age.

### Statistical analyses

All analyses were performed using SPSS 26.0 statistical software. The descriptive statistics included baseline clinical information, clinical risk factors, FRAX scores, and other outcomes of interest. The data were tested for normal distribution. Continuous variables were presented as mean ± standard deviation and were compared by analysis of variance (ANOVA) between the groups. Categorical variables were presented as frequency and percentage and compared with the chi-square test. To compare the baseline characteristics between adult patients with postoperative DTC and control groups, we used the Mann–Whitney *U* test for continuous variables and the Chi-squared test or the Fisher’s exact test for categorical variables. The test method of grouping according to TSH level is the same as above. For the prediction of osteoporosis (BMD T score ≤ 2.5 was used as the gold standard), the receiver operating characteristic curve (ROC) of the FRAX score was employed to determine the optimal cutoff values using the Youden index. All statistical data are presented as the mean (95% confidence interval (CI)). *P* < 0.05 indicates statistically significant.

## Results

### Baseline characteristics

In a population of 64 patients with TSH suppression, according to the T score, the patients were divided into three groups, including 24 (37.5%) individuals with normal bone mass (T score of ≥ −1), 23 (35.9%) individuals with osteopenia (T score of −2.5~−1), and 17 (26.6%) individuals with osteoporosis (T score of ≤ −2.5). Compared to individuals with a T score of ≥ −1, those with a T score of ≤ −2.5 tend to have a higher risk of fracture and were more likely to be older (54.35 years vs. 44.38 years, *p* = 0.021), men (76.47% vs. 41.67%, *p* = 0.028), and underweight (76.47% vs. 41.67%, *p* = 0.038). Individuals with a T score of < 2.5 had longer postoperative time than those with a T score of > −1 (3.22 years vs. 2.76 years, *p* = 0.646) (**Table 1**).

**Table 1:** Characteristics of the study population.

Baseline	ALL (*n* = 64)	*T* ≥ −1 (*n* = 24)	−2.5 < *T* < −1 (*n* = 23)	*T* ≤ −2.5 (*n* = 17)	*p*-value
Sex (male)	40 (62.5%)	10 (41.7%)	17 (73.9%)	13 (76.5%)	0.028 (< 0.05)
Age	48.86 ± 11.57	44.38 ± 10.38	49.48 ± 12.47	54.35 ± 9.76	0.021 (< 0.05)
Body mass index (kg/m^2^)	25.12 ± 3.82	26.62 ± 4.19	23.85 ± 3.62	24.79 ± 2.88	0.038 (< 0.05)
Prior fracture	7 (10.9%)	5 (20.8%)	1 (4.4%)	1 (5.9%)	0.150
Parental hip fracture	3 (4.7%)	2 (8.3%)	1 (4.4%)	0 (0%)	0.329
Current smoking behavior	8 (12.5%)	5 (20.8%)	2 (8.7%)	1 (5.9%)	0.291
Rheumatoid arthritis	2 (3.1%)	1 (4.2%)	0 (0%)	1 (5.9%)	0.391
secondary osteoporosis	6 (9.4%)	1 (4.2%)	2 (8.7%)	3 (17.7%)	0.354
Consume ≥ 3 units of alcohol/day	5 (7.8%)	3 (12.5%)	1 (4.4%)	1 (5.9%)	0.556
Hypertension	16 (25.0%)	5 (20.8%)	6(26.1%)	5 (29.4%)	0.812
Diabetes	10 (15.6%)	4 (16.7%)	4(17.4%)	2 (11.8%)	0.870
Coronary heart disease	4 (6.3%)	2 (8.3%)	1 (4.4%)	1 (5.9%)	0.850
Postoperative time (years)	2.70 ± 3.22	2.76 ± 3.50	2.25 ± 2.75	3.22 ± 3.51	0.646
TSH	0.22 (0.02 to 1.07)	0.26 (0.05 to 1.27)	0.25 (0.01 to 0.83)	0.04 (0.01 to 0.71)	0.760
FRAX predicted probability of MOF (without BMD)	2 (1.40 to 2.78)	1.8 (1.23 to 2.40)	2 (1.30 to 3.70)	2.5 (1.95 to 4.55)	0.036 (< 0.05)
FRAX predicted probability of MOF (with BMD)	2.4 (1.60 to 4.63)	1.6 (1.25 to 1.98)	2.4 (1.80 to 3.50)	12 (4.75 to 15.00)	0 (< 0.05)
FRAX predicted probability of MOF (with TSH)	2.8 (1.80 to 4.18)	1.95 (1.58 to 3.35)	2.9 (1.70 to 4.10)	4 (2.55 to 6.70)	0.015 (< 0.05)
FRAX predicted probability of hip fracture (without BMD)	0.2 (0.10 to 0.48)	0.2 (0.10 to 0.30)	0.2 (0.10 to 0.80)	0.3 (0.20 to 1.20)	0.022 (< 0.05)
FRAX predicted probability of hip fracture (with BMD)	0.3 (0.10 to 2.00)	0.1 (0 to 0.10)	0.6 (0.20 to 0.80)	7.7 (2.40 to 12.5)	0 (< 0.05)
FRAX predicted probability of hip fracture (with TSH)	0.5 (0.20 to 1.10)	0.2 (0.10 to 0.68)	0.6 (0.10 to 1.10)	1.1 (0.35 to 2.75)	0.007 (< 0.05)

Description of the basic information about the disease group. Data are presented as n (%), mean ± SD. FRAX without BMD means no BMD and no age scoring. FRAX with BMD represents the entry of BMD without age assignment. FRAX with TSH represents the fracture risk calculated without the inclusion of BMD but with age assignment.

In the case of matched sex ratio, age, and BMI between the disease (64 people) and control (30 people) groups, the T score of the disease group was significantly lower than that of the control group (−1.36 vs. −1.09; *p* = 0.030). Compared to the control group, the probability of MOF from FRAX and the probability of hip fracture from FRAX (with T score and with TSH) in the postoperative population of differentiated thyroid cancer is higher than that of the control group (2.40 vs. 1.60, *p* = 0.001; 2.80 vs. 2.00, *p* = 0.029; 0.30 vs. 0.15, *p* = 0.037; 0.50 vs. 0.25, *p* = 0.029; **Table 2**).

**Table 2:** Diseased group and control group.

	Diseased group (*n* = 64)	Control group person (*n* = 30)	*p*-value
Sex (female)	40 (62.5%)	18 (60.0%)	0.816
Age	48.86 ± 11.57	46.10 ± 14.17	0.318
BMI	25.14 ± 3. 82	25.96 ± 3.69	0.326
Probability of MOF from FRAX without BMD	2.00 (1.40 to 2.78)	2.00 (1.40 to 3.23)	0.884
Probability of MOF from FRAX with BMD	2.40 (1.60 to 4.63)	1.60 (1.18 to 2.40)	0.001 (<0.05)
Probability of MOF from FRAX with TSH	2.80 (1.80 to 4.18)	2.00 (1.40 to 3.23)	0.029 (<0.05)
Probability of hip fracture from FRAX without BMD	0.20 (0.10 to 0.48)	0.25 (0.10 to 0.73)	0.905
Probability of hip fracture from FRAX with BMD	0.30 (0.10 to 2.00)	0.15 (0.10 to 0.33)	0.037 (< 0.05)
Probability of hip fracture from FRAX with TSH	0.50 (0.20 to 1.10)	0.25 (0.10 to 0.73)	0.029 (<0.05)
T score	−1.36 (−2.50 to −4.45)	−1.09 (−1.45 to −0.14)	0.030 (< (0.05)

The diseased group includes patients with differentiated thyroid cancer following a thyroidectomy. The control group comprised the population without thyroid cancer. Fracture risk comparison between patients and controls. Data are presented as n (%), mean ± SD, or median (interquartile range).

### Comparison of fracture risk at different TSH levels

Herein, we compared the fracture risk from FRAX according to various algorithms and T scores among patients receiving l-thyroxine suppressive therapy for DTC with TSH of < 0.01 µIU/mL, 0.1–2 µIU/mL, and > 2 µIU/mL. There were nine patients in the TSH of < 0.1 µIU/mL group, 46 in the TSH of 0.1–2 µIU/mL group, and nine in the TSH of > 2 µIU/mL group. The results showed that the risk of MOF and hip fracture without a T score was significantly different under various TSH levels (*p* < 0.05). The risk of MOF and hip fracture with a T score was similar among the three groups. However, the risk of MOF and hip fracture after age-weighted score of TSH increased significantly with decreasing TSH levels (*p* < 0.05) (**Table 3**).

**Table 3:** Gradient of risk for incident fractures in groups stratified by TSH.

HR (95% CI)	TSH < 0.1 (*n* = 9)	TSH 0.1–2 (*n* = 46)	TSH > 2 (*n* = 9)	*p*-value
Probability of MOF from FRAX without BMD	2.70 (2.30 to 6.20)	1.90 (1.30 to 2.60)	1.90 (1.45 to 4.10)	0.031 (< 0.05)
Probability of MOF from FRAX with BMD	3.80 (2.15 to 7.95)	2.10 (1.50 to 3.93)	4.30 (1.50 to 9.55)	0.286
Probability of MOF from FRAX with TSH	6.00 (3.10 to 7.70)	2.70 (1.80 to 4.00)	1.90 (1.45 to 4.10)	0.030 (< 0.05)
Probability of hip fracture from FRAX without BMD	0.40 (0.25 to 3.15)	0.20 (0.10 to 0.33)	0.20 (0.15 to 1.25)	0.014 (< 0.05)
Probability of hip fracture from FRAX with BMD	1.00 (0.10 to 5.10)	0.3 (0.10 to 1.05	1.40 (0.10 to 5.4)	0.581
Probability of hip fracture from FRAX with TSH	2.50 (0.50 to 4.55)	0.45 (0.18 to 0.93)	0.20 (0.15 to 1.25)	0.048 (< 0.05)
T score	−1.82 (−3.58 to −0.81)	−1.22 (−2.25 to −0.39)	−1.86 (-3.13 to 0.04)	0.439

Fracture risk according to TSH level. Data are presented as median (interquartile range).

### Optimal cutoff value of FRAX score for predicting osteoporosis

**Figure 1:**
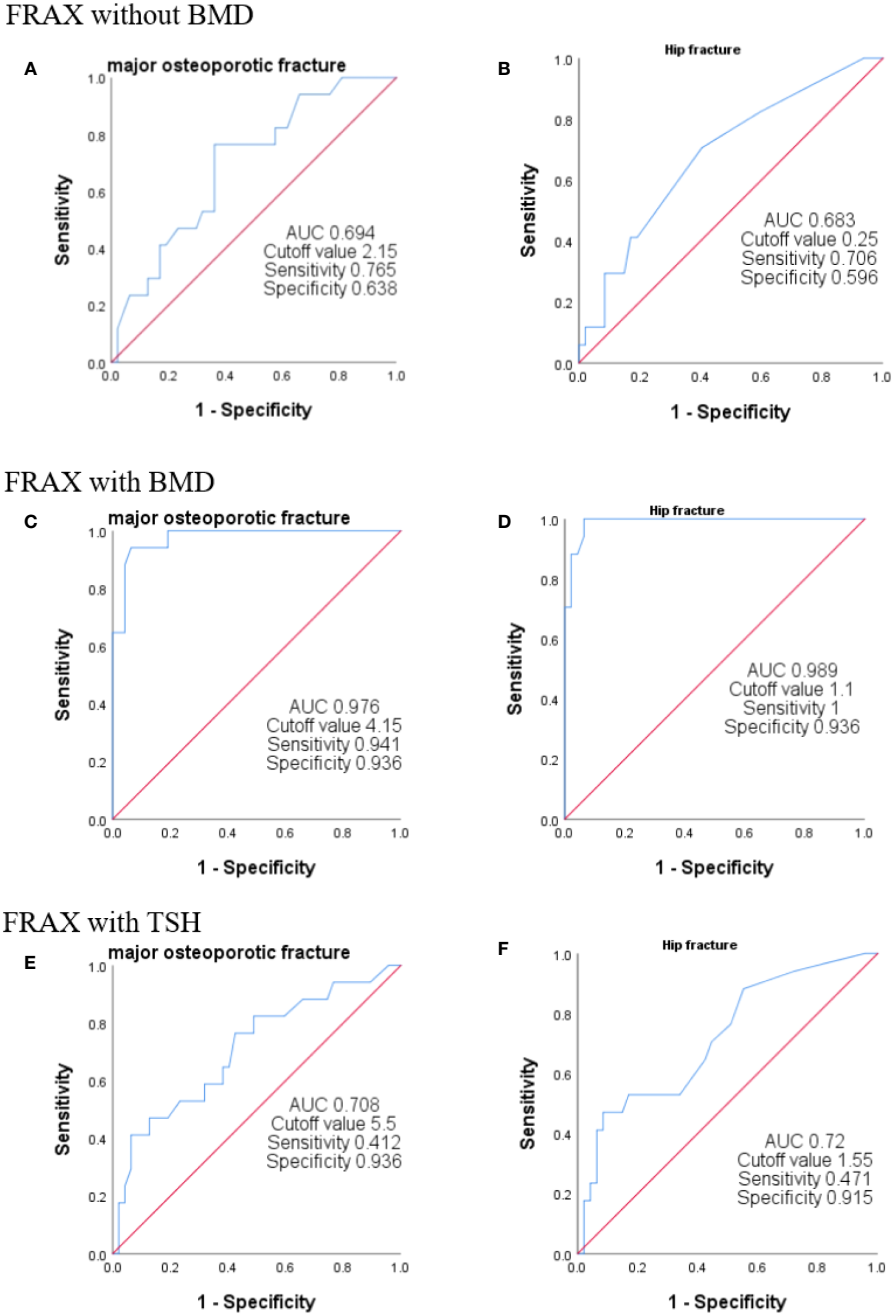
ROC curves of FRAX with different algorithms. The ROC curve was used to analyze the efficiency of FRAX score in predicting sarcopenia. We plotted the curves using *T* < 2.5 as the gold standard for the diagnosis of osteoporosis. FRAX without BMD indicates no BMD and no age scoring **(A**, **B)**. FRAX with BMD represents the entry of BMD without age assignment **(C**, **D)**. FRAX with TSH represents the fracture risk calculated without the inclusion of BMD but with age assignment **(E**, **F)**.

A ROC curve analysis was carried out to determine the optimal cutoff values of the FRAX score for major osteoporotic or hip fractures (**Figure 1**). In the FRAX without BMD, the area under the curve (AUC) values of major osteoporotic or hip fractures were 0.694 (95% CI = 32.5–54.8) and 0.683 (95% CI = 28.4–50.1), respectively (**Figures 1A, B**), and the cutoff values for predicting major osteoporotic fracture and hip fracture were 2.15% and 0.25%, respectively, with a sensitivity, specificity, positive predictive value (PPV), and negative predictive value (NPV) of 0.765 and 0.706, 0.638 and 0.596, 0.433 and 0.387, and 0.882 and 0.848, respectively. Similarly, in the FRAX with BMD, the AUC values of major osteoporotic or hip fractures were 0.976 (95% CI = 63.9–94.1) and 0.989 (95% CI = 65.5–94.4), respectively (**Figures 1C, D**), and the cutoff values were 4.15% and 1.1%, respectively, with the sensitivity, specificity PPV, and NPV as 0.941 and 1, 0.936 and 0.936, 0.842 and 0.85, and 0.978 and 1, respectively. Finally, in the FRAX with TSH, the AUC values of major osteoporotic or hip fractures were 0.708 (95% CI = 40.5–88.9) and 0.72 (95% CI = 40.8–85.3), respectively (**Figures 1E, F**). The cutoff values were 5.5% and 1.55%, respectively, with the sensitivity, specificity, PPV, and NPV at 0.412 and 0.471, 0.936 and 0.915, 0.7 and 0.667, and 0.815 and 0.827, respectively (**Table 4**).

**Table 4:** Comparison of forecast parameters of different calculation methods.

	AUC	Cutoff value	Sensitivity	Specificity	PPV	NPV
Probability of MOF from FRAX without BMD	0.694	2.15	0.765	0.638	0.433	0.882
Probability of MOF from FRAX with BMD	0.976	4.15	0.941	0.936	0.842	0.978
Probability of MOF from FRAX with TSH	0.708	5.5	0.412	0.936	0.7	0.815
Probability of hip fracture from FRAX without BMD	0.683	0.25	0.706	0.596	0.387	0.848
Probability of hip fracture from FRAX with BMD	0.989	1.1	1	0.936	0.85	1
Probability of hip fracture from FRAX with TSH	0.72	1.55	0.471	0.915	0.667	0.827

## Discussion

The present study demonstrated that patients treated with TSH suppression following thyroidectomy had a 0.27-fold decline in BMD T score and a higher fracture risk compared to the matched controls. Both PMOF with BMD and PMOF after TSH assignment increased by 0.8%. These results suggested that TSH suppression has adverse effects on BMD and fracture risk in postoperative DTC patients. When the patients were grouped according to TSH level, the lower the TSH level, the higher the fracture risk without BMD, and the higher the TSH level. The ROC operating curve indicated that the FRAX score with TSH level also improved the diagnostic efficiency of osteoporosis compared to the fracture risk without BMD. The FRAX by TSH weighted is convenient and quick, making it ideal for rapid clinical assessment of a patient’s risk of osteoporotic fracture without waiting for BMD results in order to facilitate early intervention treatment of osteoporotic fractures in a cost-effective manner.

The current findings provide additional evidence of the association between TSH suppressive therapy and osteoporosis. The study by Lin et al. showed a strong dose–response and duration–response correlation between levothyroxine use and osteoporosis risk in patients with differentiated thyroid cancer (12). Moreover, Heijckmann et al. showed that patients with well-differentiated thyroid cancer do not have an increased risk of low bone mass or an increased incidence of vertebral fractures, at least when they were treated with relatively low doses of levothyroxine (16). Our data proved that DTC patients treated with levothyroxine had an increased risk of osteoporosis, and the postoperative time was 2.70 years ± 3.22 years. However, T scores were not affected by TSH, no significant difference was detected in the hazard ratio among TSH groups, and no linear trend was observed, based on the association between the dosage and duration of medication, i.e., TSH suppression therapy. However, the fracture risk calculated by the TSH-weighted FRAX score was significantly higher than that calculated by the other two methods.

In addition to the risk factors currently included in FRAX, previous studies have shown that type 2 diabetes mellitus and previous falls should be considered independent risk factors in predicting MOF and HF (17–20). The present study consisted of concomitant diabetes in each group of patients, which may have some effects on BMD; however, we have tried to control the duration of diabetes in patients within 1 year to reduce the error. To date, no study has shown whether the TSH level of postoperative patients with differentiated thyroid cancer can be included in the risk factors of the FRAX tool. To date, whether the TSH level of postoperative patients with DTC can be included in the risk factors of the FRAX tool needs further study. A previous study has shown that high levels of TSH are positively correlated with BMD of the femoral neck and lumbar spine (21). Similarly, some clinical studies suggested that low-normal TSH is independently associated with bone mass loss, increased bone turnover, and decreased BMD in both men and postmenopausal women (22–24). The direct effects of TSH on bone remodeling, osteoblast bone formation, and osteoclast bone resorption are mediated by the TSH receptor (TSHR) in osteoblasts and osteoclast precursors (25, 26). This phenomenon has also been demonstrated in animal experiments, wherein TSH from the anterior pituitary inhibits bone resorption by osteoclasts. Accumulating evidence indicated that TSH is closely related to osteoporosis. Based on our results, clinicians assessing the risk of fracture in postoperative DTC patients should adopt TSH-weighed age in FRAX calculation according to our estimation method (when BMD is unknown) to account for the independent effect of TSH on MOF and HF. Nonetheless, larger studies are needed to examine whether TSH is an independent factor in improving FRAX prediction of fracture risk.

Interestingly, our study has crucial clinical implications. We speculated that the FRAX score could be applied to guide physicians’ treatment decisions in the treatment of osteoporotic patients with DTC after postoperative TSH suppression therapy. Due to the differences in national conditions and epidemiology, the intervention thresholds based on the cost-efficiency of FRAX score differ across countries (27–31). However, a few studies on the FRAX-based osteoporosis intervention threshold have been conducted in China. A prospective cohort study of postmenopausal women at Peking Union Medical College Hospital, Chinese Academy of Medical Sciences, Beijing, China, showed that for Chinese postmenopausal women, maximal clinical and economic benefits could be achieved when the FRAX intervention threshold is set at 7% (32). The ROC curve revealed that the specificity of the FRAX after TSH weighting (without BMD) was higher than that of the FRAX without a score (PMOF: 0.936 vs. 0.638, PHF: 0.915 vs. 0.596), which was close to the FRAX with only BMD (PMOF: 0.936, PHF: 0.936), but the sensitivity was slightly low (PMOF: 0.412, PHF: 0.471). The FRAX cutoff value after TSH scoring was also the highest among the three groups; therefore, when used as the diagnostic criteria of osteoporosis, it will reduce the rate of misdiagnosis. The FRAX improved the accuracy and specificity of predicting fracture risk without BMD and provided a foundation for subsequent intervention and FRAX-based intervention thresholds for maximum clinical benefit in the DTC population.

In summary, our findings provide additional evidence that TSH suppression therapy decreases BMD in postoperative patients with DTC. The FRAX tool is suitable for postoperative DTC patients receiving TSH suppression therapy, and the TSH-weighed FRAX can improve the accuracy of osteoporosis diagnosis without BMD. Nevertheless, the present study has some limitations. Some patients have a brief medication duration (< 1 year) and a short duration of TSH suppression therapy, but there is no significant impact on bone density; however, they may reach a high bone density. The treatment of DTC patients with osteoporosis using active vitamin D, bisphosphonate, calcitonin, estrogen, vitamin K, recombinant human parathyroid hormone, and deslizumab needs further investigation. Whether TSH can be included in FRAX as an independent risk factor for 10-year fracture risk needs further research.

### Scope statement

Through investigation, we learned that the frontiers of the Journal of Endocrinology’s main research areas include all areas of endocrinology, including thyroid disease in the field of endocrinology. The field of thyroid mainly includes the latest research in the field of thyroid diseases and thyroid cancer.

We further confirmed the relationship between the level of TSH suppression and the risk of osteoporosis and fracture in patients treated with TSH suppression after thyroid cancer surgery.

Secondly, the purpose of our study is to develop a more suitable osteoporosis assessment tool for the TSH-suppressed in postoperative patients with thyroid cancer. Through the exploration of different levels of TSH values, the age-weighted scoring method of the FRAX tool (a fracture risk assessment tool) is used to more accurately evaluate the long-term risk of osteoporosis in this population. Your journal also contains many studies on thyroid cancer, but there are few studies on the relationship between thyroid cancer and osteoporosis. In our study, after many attempts, a FRAX cardiac model with high specificity was finally selected to provide greater guiding significance for the subsequent treatment of osteoporosis in patients with thyroid cancer after TSH suppression therapy.

## Data availability statement

The original contributions presented in the study are included in the article/supplementary material. Further inquiries can be directed to the corresponding author.

## Ethics statement

The study was approved by the Institutional Review Board of PLA 960th Hospital [(2022) Research Ethics (45)]. All procedures involving human participants were in accordance with the ethica standards of the institutional and/or national research committee and with the 1964 Helsinki Declaration and its later amendments or comparable ethical standards. Informed consent was obtained from all participants in the study.

## Author contributions

HJ: Formal Analysis, Writing – original draft. WQ: Formal Analysis, Writing – original draft, Writing – review & editing. XC: Data curation, Formal Analysis, Writing – original draft. ML: Formal Analysis, Writing – original draft. YQ: Investigation, Writing – review & editing. ZJ: Methodology, Writing – review & editing. ZZ: Methodology, Writing – review & editing.
